# Adult fibrosarcoma: From clinical challenges to cutting-edge innovations

**DOI:** 10.18632/oncoscience.624

**Published:** 2025-09-04

**Authors:** Reshmi Sultana, Suryadevara Sailaja

**Affiliations:** ^1^Department of General Surgery, Senior Resident, All India Institute of Medical Sciences, Bibinagar, Hyderabad, Telangana 508126, India; ^2^Department of Surgical Oncology, Consultant Surgical Oncologist, Alluri Sitaramaraju Academy of Medical Sciences, Eluru, Andhra Pradesh 534005, India

**Keywords:** soft tissue sarcoma, thigh swelling, immunotherapy, chemotherapy, aggressive tumor

## Abstract

Adult fibrosarcoma is a rare and aggressive soft tissue malignancy originating from spindle-shaped fibroblasts, primarily affecting deep soft tissues in the extremities, trunk, head, and neck. Surgical resection with R0 margins remains the gold standard, with adjuvant radiation therapy recommended for large or high-grade tumors to reduce recurrence risk. Chemotherapy and immunotherapy play complementary roles in disease management, with emerging strategies targeting matrix metalloproteinases and tumor microenvironment interactions to enhance chemosensitivity and overcome drug resistance. Despite advances in treatment, prognosis remains poor due to the tumor’s aggressive nature. However, early detection with complete surgical excision and multimodal therapies offer the best prospects for improved patient survival.

## INTRODUCTION

Adult fibrosarcoma is a rare and aggressive soft tissue malignancy, comprising only about 1–3.6% of adult sarcomas. It originates from spindle-shaped fibroblasts and primarily affects deep soft tissues in the extremities, trunk, head, and neck, with rare occurrences in the ovary or trachea.

Defined by the World Health Organization as a malignant neoplasm of fibroblasts with variable collagen production and a characteristic “herringbone” pattern, adult fibrosarcoma was once considered the most common sarcoma in adults. However, its incidence has sharply declined due to advancements in soft tissue tumor classification, the identification of distinct fibrosarcoma subtypes, and improved understanding of mesenchymal and non-mesenchymal tumors that mimic fibrosarcoma [1]. Fibrosarcoma can be categorized into two distinct types: infantile fibrosarcoma and adult-type fibrosarcoma. Unlike the infantile form, which the World Health Organization classifies as an intermediate malignancy with a low tendency to metastasize, adult fibrosarcoma is recognized as a highly aggressive and malignant tumor [2].

## CASE REPORT

A 30-year-old female patient presented with a progressively enlarging swelling in the upper anterior region of the right thigh, which had been present for the past three months. The swelling developed insidiously and gradually increased in size until it reached its current dimensions. The patient also reported a dull, aching pain in the right lower limb, which began approximately one month ago and has persisted.

Upon clinical examination, a single, firm, oval-shaped swelling measuring approximately 8 × 6 cm was palpated in the anterior aspect of the proximal one-third of the right thigh. The swelling was non-pulsatile and immobile, with no evidence of fluctuation or tenderness. The overlying skin appeared normal in texture and color, with no signs of ulceration or inflammation, and remained pinchable upon examination. Notably, there was no involvement of inguinal lymph nodes.

**Figure 1:**
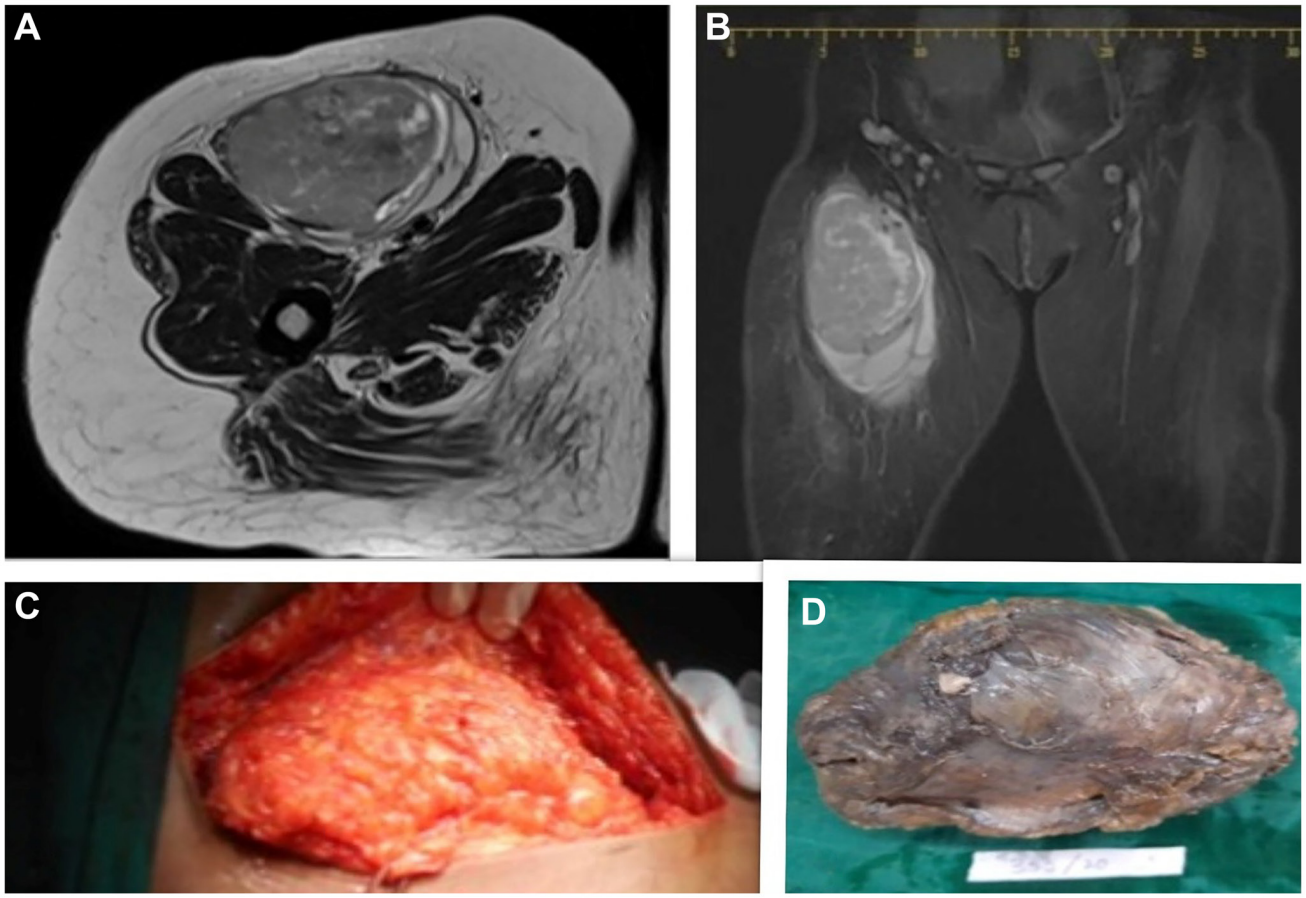
(**A**, **B**) Axial and coronal sections of MRI showing a well-encapsulated, heterogeneous lesion measuring approximately 12 × 7.5 × 6 cm, originating from the belly of the sartorius muscle. (**C**) Intra-op image of the lesion. (**D**) Excised specimen.

MRI findings indicated a well-encapsulated, heterogeneous lesion measuring approximately 12 × 7.5 × 6 cm, suggestive of malignant fibrous histiocytoma originating from the belly of the sartorius muscle (Figure 1). A Trucut biopsy further suggested the presence of a spindle cell neoplasm.

Following these diagnostic findings, the patient underwent a wide local excision of the tumor. The excised specimen was subsequently sent for histopathological examination and immunohistochemistry (IHC), which confirmed the final diagnosis as adult fibrosarcoma, classified as Grade 2. Patient had also received external beam radiotherapy postoperatively.

## DISCUSSION

### Histologic architecture

Adult fibrosarcoma is a diagnosis of exclusion and demonstrates a hallmark histologic architecture, with monomorphic spindle-shaped fibroblasts aligned in a distinctive herringbone pattern embedded within a variably collagenous matrix. Tumor cells possess scant cytoplasm, elongated hyperchromatic nuclei, and exhibit frequent mitotic activity, particularly pronounced in higher-grade lesions. Despite this proliferative profile, nuclear pleomorphism remains limited, a feature that aids in distinguishing fibrosarcoma from other aggressive spindle cell sarcomas [3].

### Molecular alterations

Adult fibrosarcoma is marked by chromosomal instability, often presenting with aneuploidy and recurrent NTRK3 gene fusions (*STRN1-NTRK3* or *STRN3-NTRK3*), which are actionable targets for tyrosine kinase inhibitors. Critical amplifications in chromosome 12q (especially 12q14–q15 and 12q21) and gains in regions like 1p21, 4q31.3, 7p21, and Xp22.1–p22.2 are common and tied to poor prognosis [4]. These molecular alterations help differentiate fibrosarcoma from other spindle cell neoplasms and inform potential targeted treatment strategies. TP53, CDKN2A, and RB1 are the most frequently altered genes in soft tissue sarcomas, with TP53 mutations and CDKN2A/RB1 copy number losses playing pivotal roles in tumor progression and prognosis. Among these, CDKN2A loss stands out for its strong association with early recurrence and poor outcomes, especially in fibrosarcoma and MPNST [5].

### Tumor-host neurobiology

Adult fibrosarcoma is a locally aggressive malignancy with a high propensity for hematogenous spread, particularly in high-grade variants that exhibit rapid proliferation, necrosis, and recurrence. Adult fibrosarcoma leverages a sophisticated neurobiological strategy to reshape systemic homeostasis in favor of tumor growth. Beyond evading immune surveillance, it actively manipulates neuroendocrine pathways through the secretion of cytokines, catecholamines, serotonin, melatonin, glucocorticoids, and other mediators. These tumor-derived signals target key regulatory centers i.e., the hypothalamus, pituitary, adrenal glands, and thyroid and disrupts host balance via feedback mechanisms that support tumor expansion [6].

Simultaneously, fibrosarcoma engages in reciprocal signaling with autonomic and sensory nerves within the tumor microenvironment. This bidirectional crosstalk fosters neural infiltration and neoaxonogenesis, while neurotransmitters released from infiltrating nerves drive tumor proliferation, angiogenesis, and immune suppression. The result is a remodeled tumor niche and systemic environment primed for cancer dominance. Extending beyond local effects, this interaction impacts brain function, circadian rhythm, stress response, and immune dynamics mirroring mechanisms seen in other cancers that hijack neuroimmune and neuroendocrine systems [6, 7].

Collectively, adult fibrosarcoma exemplifies how solid tumors can orchestrate a multi-layered physiological reprogramming, warranting therapeutic strategies that address both tumor-intrinsic mechanisms and host regulatory networks.

### Imaging features

MRI reveals well-defined lesions with hypointense signals on T1WI and heterogeneous intensity on T2WI, which are more prominent on fat-saturated sequences. CT imaging shows iso-attenuating masses with band-like areas, resembling muscle tissue. Post-contrast scans with Gd-DTPA highlight characteristic enhancement patterns, including heterogeneous peripheral enhancement and a spoke-wheel-like configuration, strongly indicative of adult fibrosarcoma [8]. PET-CT assists with the metastatic work-up.

### Immunohistochemistry and sub-types

Immunohistochemically, the tumor exhibits diffuse vimentin positivity and a high Ki-67 proliferative index, consistent with a mesenchymal origin and active mitosis. The absence of markers such as S100, cytokeratin, and smooth muscle actin helps exclude other spindle cell neoplasms, supporting a fibroblastic lineage. Advanced immunohistochemical and molecular techniques are essential for accurately classifying fibrosarcoma subtypes, which often display overlapping morphologic and genetic features.

Key subtypes include Low-Grade Fibromyxoid Sarcoma, Sclerosing Epitheloid Fibrosarcoma, and Myxofibrosarcoma. Additionally, differential diagnosis should carefully consider spindle-cell neoplasms such as Solitary Fibrous Tumor, Monophasic Fibrous Synovial Sarcoma, Epitheloid Sarcoma, spindle cell variants of Angiosarcoma, Malignant Peripheral Nerve Sheath Tumor, and Aggressive Fibromatosis [1, 9].

### Grading and staging systems

AJCC Grading helps stage the disease and predict tumor behavior and guide treatment. The FNCLCC system assigns scores based on differentiation, mitotic count and tumour necrosis where each component is scored from 1–3. Total of these scores is further divided into grades as follows:

Grade 1 (G1): Score 2–3 → Low-grade, slower growth and spread.
Grade 2 (G2): Score 4–5 → Intermediate-grade.
Grade 3 (G3): Score 6–8 → High-grade, aggressive behavior.
Grade X: Grade cannot be assessed.

AJCC Staging is determined by size of the tumour (T), nodal spread, grade determined by FNCLCC and metastasis [10]. Staging is done as shown in Table 1.

**Table 1:** Staging for adult fibrosarcoma

Stage IA	Tumour size <5 cms (T1) Grade 1 or Grade X No nodal spread or metastasis
Stage IB	Tumour size 5–10, 10–15, >15 cms (T2, T3, T4) Grade 1 or Grade X No nodal spread or metastasis
Stage II	Tumour size <5 cms (T1) Grade 2 or Grade 3 No nodal spread or metastasis
Stage III A	Tumour size 5–10 cms (T2) Grade 2 or Grade 3 No nodal spread or metastasis
Stage III B	Tumour size 10–15 cms, >15 cms (T3, T4) Grade 2 or Grade 3 No nodal spread or metastasis
Stage IV	Tumour of any size, any grade with either nodal spread or metastasis.

### Treatment strategies

The surgical approach for adult fibrosarcoma is guided by the tumor’s location, size, and grade. Whenever feasible, wide resection with R0 margins is performed to achieve complete tumor removal. For tumors exceeding 5 cm, deeply situated, or classified as high-grade, adjuvant radiation therapy is recommended to minimize recurrence risk [11].

Though, adult fibrosarcoma is chemoresistant, chemotherapy can be administered in the neo-adjuvant setting in high-grade or metastatic fibrosarcomas, with commonly used agents including doxorubicin, ifosfamide, gemcitabine, and paclitaxel [12, 13]. Stage-wise management of Adult Fibrosarcoma is briefly outlined in Table 2.

**Table 2:** Stage- wise management

Stage	Description	Treatment options
Stage I	Localised low grade tumour, no nodal or distant metastasis	Wide local resection with R0 resection (Usually, 2 cm margin is recommended but no strict guidelines.) Re-resection if possible in R1/R2 resections. Adjuvant RT in deeply situated tumors or size >5 cm.
Stage II	High grade tumour <5 cm, no nodal or distant metastasis	Wide local resection with R0 resection (Usually, 2 cm margin is recommended but no strict guidelines). Re-resection if possible in R1/R2 resections. Adjuvant RT and chemotherapy considered.
Stage III	High grade tumour >5 cm, no nodal or distant metastasis	Neo-adjuvant chemotherapy considered. Wide local resection with R0 resection wherever feasible. Re-resection if possible in R1/R2 resections. Adjuvant RT to prevent local recurrence.
Stage IV	Nodal or distant metastasis present	Debulking large or symptomatic lesions to alleviate pain, bleeding, or obstruction. Metastasectomy for isolated pulmonary metastasis in selected patients. EBRT /SBRT when surgery isn’t feasible. Chemotherapy, immunotherapy and targeted therapy play significant role.

### Advances in treatment

Immune checkpoint inhibitors like pembrolizumab and nivolumab enhance immune system activity by targeting the PD-1/PD-L1 pathway, potentially improving treatment response [14].

In tumors harboring NTRK3 fusions (e.g., STRN1-NTRK3), larotrectinib or entrectinib can achieve durable responses and are considered in molecular-guided approaches [15].

Advances in targeting matrix metalloproteinases (MMPs) and tumor microenvironment interactions have opened new avenues for fibrosarcoma treatment, focusing on selective inhibition and localized therapeutic strategies. Innovations like TIMP-1-GPI application and homotrimeric collagen degradation show promise in enhancing chemosensitivity and overcoming drug resistance, potentially improving patient outcomes [2].

Although not yet standard in fibrosarcoma, further targeting neural-tumor interactions (e.g., anti-NGF therapies, β-blockers, Trk inhibitors) is a growing area of interest in oncology and represent a promising frontier in cancer treatment, offering novel avenues to disrupt tumor-supportive neural signaling and reshape the tumor microenvironment [7].

## CONCLUSION

Adult fibrosarcoma is a rare and highly aggressive malignancy requiring precise diagnosis and multimodal treatment. While prognosis remains poor, complete surgical excision with R0 margins and effective adjuvant therapies offer the best chance for disease control and improved outcomes.
